# A Cross‐Sectional Study to Assess the Prevalence of Cognitive Impairment and Its Associated Factors Among the Elderly in Kaniyambadi Block, Vellore

**DOI:** 10.1002/puh2.70110

**Published:** 2025-08-15

**Authors:** Manoj Jacob Dhinagar, Vinod Joseph Abraham, Zacharia Mathew

**Affiliations:** ^1^ Department of Community Medicine Christian Medical College Vellore India; ^2^ Department of Internal Medicine Christian Medical College Vellore India

**Keywords:** cognitive impairment, dementia, depression, elderly, rural India

## Abstract

**Aims:**

This study aims to determine the prevalence of mild cognitive impairment and major neurocognitive disorder among adults aged greater than or equal to 60 in Kaniyambadi block, Vellore, and the factors associated with cognitive impairment.

**Settings and Design:**

A community based cross sectional study was conducted on 360 adults greater than or equal to the age of 60 residing in Kaniyambadi block, Vellore.

**Methods and Material:**

A semi‐structured interviewer‐based questionnaire was administered to the participant. Their subjective and objective cognitive abilities were assessed along with their ability to perform their activities of daily living. The participants were also screened for depression.

**Statistical Analysis Used:**

Univariate analysis was done using measures of central tendencies and proportions. Bivariate analysis was done using Chi square test, and logistic regression was also performed.

**Results:**

The prevalence of mild cognitive impairment among adults aged more than or equal to 60 residing in Kaniyambadi block was 20% (95% CI 15.9–24.5). The prevalence of major neurocognitive disorder in the same population was 4.4% (95% CI 2.5–7.1), and the prevalence of depression was 18.9% (95% CI 14.9–23.3). Age greater than or equal to 70 (AOR 2.24 [1.38–3.64]), no formal education (AOR 2.62 [1.52–4.48]), and depression (AOR 3.64 [1.90–6.99]) were found to be statistically significantly associated with cognitive impairment.

**Conclusions:**

The overall prevalence of mild cognitive impairment and major neurocognitive disorder in Kaniyambadi block was found to be similar to the prevalence in other parts of the nation. Adults aged more than 70 and those with no formal education are associated with a greater risk of developing cognitive impairment. As depression is also associated with cognitive impairment, it is imperative to screen the elderly with depression and other psychiatric illnesses for cognitive impairment.

**Key Messages:**

Cognitive impairment is an emerging public health problem that is affecting many elderly people in the population. Early identification and cognitive rehabilitation of those affected with mild cognitive impairment can help slow the progress to major neurocognitive decline.

## Graphical abstract

Cognitive impairment is an emerging public health problem. A study done in rural Tamil Nadu, India showed the prevalence of mild cognitive impairment to be 20% and major neurocognitive disorder 4.4%. Further studies are needed to ascertain the causes behind cognitive impairment and develop strategies to mitigate it.

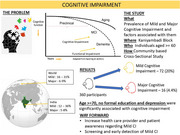

## 1 Introduction

Cognitive impairment is an emerging concept that has entered public discourse during the latter half of the 20th century. There has been increased interest in identifying the boundary between the low functioning normal ageing adult and the onset of dementia. Cognitive impairment refers to a decline in the ability to think and process information. This can include difficulties with memory, problem‐solving, attention, and other mental processes [1]. Cognitive impairment can be caused by a variety of factors, including brain injury, stroke, aging, or certain medical conditions. It can range from mild to severe and can affect a person's ability to function independently in daily life.

Mild cognitive impairment (MCI) is a syndrome characterized by cognitive decline greater than expected for an individual's age and level of education but which does not significantly interfere with daily activities [2]. Individuals with MCI may have trouble remembering recent events or names but can still perform routine tasks and take care of themselves.

Some people with MCI appear to remain stable or may return to normal over time, but within 5 years, more than half seem to develop dementia. Thus, MCI can be considered a risk factor for dementia, and its identification could lead to secondary prevention by controlling risk factors like vascular disease and hypertension among others.

When acquired cognitive impairment becomes severe enough to impair social and/or occupational functioning, dementia is typically diagnosed. Although the term dementia was used commonly in common parlance and in medical literature, it is now referred to as major neurocognitive disorder (MNCD). The DSM‐5 diagnostic criterion for MNCD, which corresponds to dementia, is the presence of substantial impairment in one or (typically) more cognitive domains. The impairment must be severe enough to impede independence in daily activities, which differentiates MNCD from MCI, where independence in daily activities is retained by the individual [3].

Neurocognitive disorders, particularly MNCDs, have devastating effects on patients, their families, the health care system, and the economy [4]. The costs of health care and uncompensated caregiving for individuals with dementia are high and increasing. Additionally, family caregivers experience elevated levels of emotional stress, depression, and health issues as a result of providing care for individuals with cognitive impairment.

Despite being a public health problem, cognitive impairment is difficult to screen at the community level due to the paucity of tools available for rural, low literacy populations. The current tools most commonly used are not available in all Indian languages, are difficult to administer, and can give falsely low scores in those with low or no formal education [5].

Given the emerging recognition of cognitive impairment as a public health concern, any forward step in our understanding of cognitive impairment is essential in further improving these strategies to alleviate the impact of cognitive impairment. There have been studies done to show that strategies like folic acid supplementation or exercise regimens in the elderly could help to improve cognitive function in the elderly with cognitive impairment [6, 7].

This study aims to assess the prevalence of cognitive impairment, both MCI and MNCD, to determine the prevalence of depression among adults aged 60 years and above and the factors that are associated with cognitive impairment among the adults aged 60 or above who are currently living in Kaniyambadi block, Vellore, South India.

## 2 Subjects and Methods

The study was conducted in Kaniyambadi block, which is a revenue block located in Vellore district, Tamil Nadu. The block is predominantly rural and consists of 88 villages with a population of about 120,000 people. The study was a community‐based cross‐sectional study carried out between April 2021 and June 2022. The study included adults aged over 60 years and excluded any adults previously diagnosed with any psychiatric illness.

The sample size was calculated using the standard formula for sample size calculation for cross sectional studies using prevalence of MCI among the elderly, found to be 8.8% in a previous study done in a similar setting [8], and an absolute precision of 3. The required sample size was 357, and the sample size attained was 360.

Multi‐stage cluster sampling method was selected as the sampling method for this study. In the first stage, 18 individual villages in Kaniyambadi block were selected as clusters using probability proportional to size (PPS). In the second stage, 20 individuals fitting the inclusion criteria from each of the selected villages were selected by simple random sampling. A total of 360 adults above the age of 60 were selected using a table of random numbers and listed in order. A refusal rate was 2.2% was encountered during the study.

The study tools used included a questionnaire regarding the demographic, socio‐economic, comorbidity, living status, and habits of the participant, AD8 questionnaire, Montreal Cognitive Assessment (MoCA), Everyday Abilities Scale for India (EASI), and Geriatric Depression Scale‐15 (GDS‐15). As per the DSM‐5 criteria, to classify an individual as mild neurocognitive disorder or MNCD, there needs to be a subjective and objective decline in the cognitive capabilities of the individual. A subjective decline was considered a decline in cognitive capability as identified by the individual themselves or a caretaker. An objective decline was considered a decline in cognitive capability of an individual as identified by a standardized cognitive testing tool. In this study, the AD8 Dementia Screening tool was used for subjective estimation of an individual's cognitive function [9, 10]. The AD8 consists of eight questions with the answers being either Yes/No. The cut‐off used in this study was 0–1: normal cognition, 2 or greater: cognitive impairment is likely to be present.

For the objective assessment of cognitive impairment, the MoCA‐T test was used. The MoCA test was validated as a tool for the detection of MCI in 2005. It was found to have a sensitivity of 90% in detecting MCI and was more sensitive than the mini–mental state examination (MMSE), which is the most widely used tool for cognitive assessment [11, 12]. The MoCA also eliminates the high ceiling effects and educational bias of the MMSE [13]. The domains tested by MoCA include visuospatial/executive, naming, attention, language, abstraction, and delayed recall assessed by the memory index score (MIS) and orientation. The maximum possible score is 30, and an extra mark is given if the participant has less than or equal to 12 years of education. The MoCA‐T was also tested in Tamil Nadu in 2022 and found to have an optimal cutoff of 24, which accommodated a sensitivity of 88.9% and a specificity of 77.9% [14]. In this study, a score of 24 or more was used to imply no cognitive impairment, whereas a score of 23 or less implied the presence of cognitive impairment. The MoCA‐T is administered in Tamil and replaces the original questions with more culturally appropriate ones for use in Tamil Nadu and other Tamil speaking areas. It was hence used in this study, as it was thought to be the most appropriate tool in this cultural, linguistic, and educational setting where this study was conducted.

Activities of daily living (ADL) was assessed using the EASI tool [15]. A cut‐off of 3 or more was used to indicate impaired ADL. Depression was screened in this study using the GDS‐15. This short‐form version has been found to have a sensitivity of 80% and specificity of 75% with a cutoff of 5/6 [16]. The Tamil version has been validated in Puducherry and has a similar sensitivity [17]. A score of 1 was given for each answer suggesting depression, and a cut‐off of 5 or more suggested the presence of depression in the participant.

Presence of subjective and objective cognitive decline without any impairment of ADL was considered positive for MCI. Presence of subjective and objective cognitive decline with impairment of ADL was considered MNCD.

The researchers visited the household of each eligible individual as in the list and interviewed the selected participant and their primary caregiver, if available or needed. An auxiliary health worker accompanied the investigator during these interviews to help develop rapport with the participants and assist in establishing a favorable environment prior to administering the questionnaire. Informed consent was obtained from the study participant after being provided the requisite information regarding the study. If the caregiver was available, the information regarding the study was provided to them as well. The study questionnaire was then administered by the investigator.

Univariate analysis was performed by calculating frequencies and percentages for variables such as socio‐demographic details, living status, income, family members, primary caregiver, comorbidities, history of head injury, family history of cognitive impairment or diagnosed psychiatric illness and substance abuse.

Bivariate analysis was done using the chi‐square test and odds ratio to determine any statistically significant association and the strength of the associations between cognitive impairment and the various factors studied, respectively. Multivariable analysis was also performed between cognitive impairment and the factors found to be significant in the bivariate analysis.

## 3 Results

A total of 360 participants were interviewed for this study. The baseline characteristics of the 360 participants who were included in the analysis for this study are included in Table 1. The mean age of the participants was 68.4, and median age was 68. The most common self‐reported comorbidity among the participants was hypertension (36.9%), followed by diabetes mellitus (31.9%). Overall, 12.8% of the participants suffered from vascular disease, which included previous cerebrovascular accidents, current cardiovascular disease or peripheral vascular disease. Commonest substance addictions mentioned by the participants included beedi (39 participants, 10.8%), alcohol (13 participants, 3.6%), and chewing tobacco (7 participants, 1.9%). The median duration of substance use was 30 years. Overall, 56% of the participants are currently using any of these substances.

**TABLE 1:** Baseline characteristics of the study population.

Sl. no.	Characteristics	Categories	Frequency (%)
1.	Age	60–64	112 (31.1)
		65–69	94 (26.1)
		≥70	154 (42.8)
2.	Gender	Male	138 (38)
		Female	222 (62)
3.	Marital status	Currently married	194 (53.9)
		Single/Widow/Widower/Separated	166 (46.1)
4.	Current occupation status	Currently working	101 (28)
		Unemployed/Retired/Pensioner	259 (72)
5.	Socio‐economic status (BG Prasad Classification 2022)	Class I–II	110 (30.6)
		Class III–V	250 (69.4)
6.	Education status	No formal education	163 (45.3)
		Primary or middle school	147 (40.8)
		High school or higher	50 (13.9)
7.	Medical comorbidity	No comorbidity	164 (45.6)
		Comorbidity present	196 (54.4)
8.	History of head injury in participants	Head injury present	349 (97)
		Head injury absent	11 (3)
9.	History of cognitive impairment in the family of the participants	Absent	345 (95.8)
		Present	15 (4.2)
10.	History of diagnosed psychiatric illness in the participants family	Absent	356 (98.9)
		Present	4 (1.1)
11.	Substance abuse by the study participants	Substance abuse absent	302 (84)
		Substance abuse present	58 (16)
12.	Depression	Depression absent	292 (81)
		Depression present	68 (19)

Table 2 shows that out of the 360 participants, 72 (20%) has MCI, and 16 (4.4%) had MNCD. A total of 75 participants were found to have an objective cognitive decline on the basis of the MoCA scores. A total of 18 participants were found to have impaired ADL on the basis of their EASI score.

**TABLE 2:** Prevalence of cognitive impairment.

	Frequency	Percentage (95% CI)
No cognitive impairment	185	51.1 (45.8–56.3)
Only subjective cognitive decline	13	3.6 (1.9–6.1)
Only objective cognitive decline	75	20.8 (16.7–25.4)
Mild cognitive impairment	72	20 (15.9–24.5)
Major neurocognitive disorder	16	4.4 (2.5–7.1)
**TOTAL**	360	100

Table 3 shows the association between different risk factors and presence of any cognitive impairment (both mild and major). Among the 360 study participants, age greater than or equal to 70, female gender, socio‐economic classification as per BG Prasad scale belonging to Class III or lower, separated from their spouse or widowed, currently not living with a spouse, requiring a primary caregiver, having no formal education, family history of cognitive impairment, presence of vascular disease, and presence of depression were all found to be significantly associated with the presence of cognitive impairment. A multiple logistic regression analysis was carried out for all the variables found to be significant in the bivariate analysis. After adjusting for all the factors, age greater than or equal to 70 (AOR 2.24 [1.38–3.64]), no formal education (AOR 2.62 [1.52–4.48]), and depression (AOR 3.64 [1.90–6.99]) were found to be statistically significantly associated with the presence of cognitive impairment.

**TABLE 3:** Association of various factors with any cognitive impairment.

Risk factor	Category	Cognitive impairment present	Cognitive impairment absent	Crude odds ratio (95% CI)	Adjusted odds ratio (95% CI)	*p* value
1. Age	≥70	96 (62.3)	58 (37.7)	2.60 (1.69–4.00)	2.24 (1.38–3.64)	<0.001
	<70	80 (38.8)	126 (61.2)			
2. Gender	Female	127 (57.2)	95 (42.8)	2.42 (1.57–3.75)	1.47 (0.84–2.58)	0.18
	Male	49 (35.5)	89 (64.5)			
3. Socio‐economic status (SES)	Class III–V	136 (54.4)	114 (45.6)	2.08 (1.31–3.31)	1.17 (0.67–2.05)	0.57
	Class I–II	40 (36.4)	70 (63.6)			
4. Marital status	Separated/Widowed	99 (59.6)	67 (40.4)	2.24 (1.47–3.42)	1.13 (0.64–1.97)	0.67
	Married	77 (39.7)	117 (60.3)			
5. Primary caregiver	Others	34 (64.2)	19 (35.8)	2.07 (1.13–3.80)	1.45 (0.72–2.90)	0.30
	Self	142 (46.3)	165 (53.7)			
6. Living arrangements	Spouse absent	98 (59.8)	66 (40.2)	2.24 (1.47–3.43)	1.75 (0.79–3.87)	0.17
	Spouse present	78 (39.8)	118 (60.2)			
7. Education	No formal education	110 (67.5)	53 (32.5)	4.11 (2.65–6.41)	2.60 (1.51–4.43)	<0.001
	Any education	66 (33.5)	131 (66.5)			
8. History of head injury	Head injury present	7 (63.6)	4 (36.4)	1.86 (0.53–6.48)	—	0.32
	Head injury absent	169 (48.4)	180 (51.6)			
9. Presence of psychiatric illness in the family	Psychiatric illness present	1 (25)	3 (75)	0.34 (0.03–3.34)	—	0.62
	Psychiatric illness absent	175 (49.2)	181 (50.8)			
10. Cognitive impairment in the family	Cognitive impairment present	13 (86.7)	2 (13.3)	7.25 (1.61–32.64)	—	0.003
	Cognitive impairment absent	163 (47.2)	182 (52.8)			
11. Substance abuse	Substance abuse present	23 (39.7)	35 (60.3)	0.64 (0.36–1.13)	—	0.15
	Substance abuse absent	153 (50.7)	149 (49.3)			
12. Comorbidities	Present	101 (51.5)	95 (48.5)	1.26 (0.83–1.91)	—	0.27
	Absent	75 (45.7)	89 (54.3)			
13. Vascular disease	Present	29 (63.0)	17 (37.0)	1.93 (1.02–3.66)	1.62 (0.78–3.39)	0.19
	Absent	147 (46.8)	167 (53.2)			
14. Depression	Present	52 (76.5)	16 (23.5)	4.40 (2.40–8.07)	3.64 (1.90–6.99)	<0.001
	Absent	124 (42.5)	168 (57.5)			

Table 4 shows that no formal education, need for a caregiver, and family history of cognitive impairment were found to be statistically significantly associated with the presence of MNCD in this study. In a logistic regression model, presence of a caregiver (AOR 5.83 [1.39–24.38]) and presence of family history of cognitive impairment (AOR 7.94 [1.18–53.13]) were found to be statistically significantly associated with the presence of MNCD.

**TABLE 4:** Association of various factors with mild cognitive impairment or major neurocognitive disorder.

Risk factor	Category	Major neurocognitive decline	Mild cognitive impairment	Odds ratio (95% CI)	Adjusted odds ratio (95% CI)	*p* value
1. Age	≥70	13 (24.1)	41 (75.9)	3.27 (0.85–12.50)	1.61 (0.35–7.25)	0.53
	<70	3 (8.8)	31 (91.2)			
2. Gender	Female	14 (21.5)	51 (78.5)	2.88 (0.60–14.28)	—	0.22
	Male	2 (8.7)	21 (91.3)			
3. Socio‐economic status (SES)	Class III–V	15 (20.8)	57 (79.2)	3.95 (0.48–32.25)	—	0.28
	Class I–II	1 (6.3)	15 (93.8)			
4. Marital status	Separated/Widowed	11 (21.6)	40 (78.4)	1.76 (0.55–5.58)	—	0.333
	Married	5 (13.5)	32 (86.5)			
5. Primary caregiver	Others	9 (52.9)	8 (47.1)	10.28 (3.00–35.23)	5.83 (1.39–24.38)	<0.001
	Self	7 (9.9)	64 (90.1)			
6. Living arrangements	Spouse absent	12 (23.5)	39 (76.5)	2.53 (0.74–8.62)	0.99 (0.23–4.22)	0.99
	Spouse present	4 (10.8)	33 (89.2)			
7. Education	No formal education	14 (24.6)	43 (75.4)	4.71 (0.99–22.22)	2.45 (0.42–14.08)	0.31
	Any education	2 (6.5)	29 (93.5)			
8. History of head injury	Head injury present	0	3 (100)	—	—	1.00
	Head injury absent	16 (18.8)	69 (81.2)			
9. Presence of psychiatric illness in the family	Psychiatric illness present	0	1 (100)	—		1.00
	Psychiatric illness absent	16 (18.4)	71 (81.6)			
10. Cognitive impairment in the family	Cognitive impairment present	5 (62.5)	3 (37.5)	10.45 (2.18–50.07)	7.94 (1.18–53.13)	0.03
	Cognitive impairment absent	11 (13.8)	69 (86.2)			
11. Substance abuse	Substance abuse present	0	11 (100)	—	—	0.20
	Substance abuse absent	16 (20.8)	61 (79.2)			
12. Comorbidities	Present	7 (14.0)	43 (86.0)	0.52 (0.17–1.56)	—	0.24
	Absent	9 (23.7)	29 (76.3)			
13. Vascular disease	Present	4 (17.4)	19 (82.6)	0.93 (0.26–3.23)	—	1.00
	Absent	12 (18.5)	53 (81.5)			
14. Depression	Present	7 (21.2)	26 (78.8)	1.37 (0.45–4.12)	—	0.58
	Absent	9 (16.4)	46 (83.6)			

## 4 Discussion

The prevalence of MCI in this study was 20% (95% CI 15.9–24.5). As the concept of MCI is an emerging one, there is a paucity of studies looking at its prevalence in different populations. The existence of varied criteria for detection and the presence of numerous tools to screen for MCI lead to large scale variations in the prevalence of MCI. The prevalence of MCI in a study done in the out‐patient department of a secondary hospital in Vellore was 36.5%. However, this study used the MMSE and the Vellore Screening Instruments for Dementia (VSID) to screen for MCI and MNCD [18]. The 10/66 dementia research group found the crude prevalence of MCI in Vellore to be 4.3% while using the Community Screening Instrument for Dementia (CSI‐D) tool [19]. A community‐based study involving 185 participants above the age of 60 in Coimbatore, also using the MoCA‐T tool and identical cut‐offs as that which was used in the present study, found the prevalence of MCI in that population to be 35.1% [20]. The discrepancy among this and several other studies dealing with the prevalence of MCI can be attributed to the variety of tools used and the lack of standardized tools in different languages as well as the heterogeneity in the cutoffs used in the various studies and the populations in these studies.

The prevalence of dementia or MNCD in this study was 4.4% (95% CI 2.5–7.1). The prevalence of dementia in the previously mentioned study done in an out‐patient department in Vellore was 1.5% [18]. The presence of multiple diagnostic criteria also cause a wide variation in the prevalence of dementia. Different criteria have led to the prevalence of dementia to range from 0.8% to 63.2% in Kaniyambadi block, Vellore, on the basis of different tools [21].

Overall, 3.6% of the study participants were found to have only subjective cognitive decline, and 20.8% had objective cognitive decline without any subjective decline. Both groups had no restriction in ADL. A study in Kerala which used the Malayalam version of the Addenbrooke's Cognitive Examination (m‐ACE) showed the prevalence of participants with subjective cognitive decline to be 13.6% and objective cognitive decline to be 11.03% [22]. The larger numbers of participants with objective cognitive decline could be as a result of the difference in the tools used and the levels of education between the populations.

In the bivariate analysis, having no formal education was found to be significantly associated with the presence of any cognitive impairment with an odds ratio of 4.11 (2.65–6.41). This was confirmed by the logistic regression analysis, where no formal education was still significantly associated with cognitive impairment (AOR 2.62 [1.52–4.48]). This is similar to the findings from studies done in comparable settings where low levels of education were found to be significantly associated with cognitive impairment [23].

Age greater than 70 was also found to be significant in the logistic regression analysis with an adjusted odds ratio of 2.24 [1.38–3.64]. Older age was found to be significantly associated with cognitive impairment, both mild and MNCDs, in another study done in Vellore, which was mentioned above [18]. Age has been found to be a significant factor associated with greater risk of developing cognitive impairment in multiple settings, including rural areas in North India and Andhra Pradesh as well [24, 25].

Depression was found to be significantly associated with cognitive impairment in the logistic regression analysis with an adjusted odds ratio of 3.64 (1.90–6.99). The association of depression and cognitive impairment has also been well‐studied, and this finding is in line with the consensus opinions and findings regarding this association [26]. Depression was also found to be significantly associated with cognitive impairment in the Indian context in a study done in Guwahati [27].

Other factors found to be significantly associated with cognitive impairment in the bivariate analysis in this study included female gender (OR 2.42 [1.57–3.75]), socio‐economic classification as per BG Prasad scale belonging to Class III or lower (OR 2.08 [1.31–3.31]), separated from their spouse or widowed (OR 2.24 [1.47–3.42]), currently living alone (OR 2.69 [1.38–6.66]), requiring a primary caregiver (OR 2.07 [1.13–3.80]), family history of cognitive impairment (OR 7.25 [1.61–32.64]), and presence of vascular disease (OR 1.93 [1.02–3.66]). However, these factors were found to not be significant in the multivariable analysis on adjusting for covariates.

The presence of medical comorbidities like diabetes and hypertension were found to not be significantly associated with cognitive impairment, which was similar to the findings in other studies [18, 28]. The association and biological relation of diabetes and hypertension to the presence of cognitive impairment is yet to be completely ascertained, and further studies are needed.

History of previous head injury and history of psychiatric illness in the family were not found to be significantly associated with cognitive impairment in this study. This could be due to the low numbers of study participants who had history of head injury in the past or had family members with cognitive impairment.

History of smoking has been found to be positively associated with cognitive decline [29]; however, substance use was not found to be associated with cognitive impairment in this study. Multiple studies evaluating the association between alcohol and cognitive impairment have yielded inconclusive, ambiguous, and contradictory findings. Alcohol has been found to be significantly associated in certain studies done in India [22, 27], but there have been other studies showing no or even a positive effect on cognition with low‐moderate levels of alcohol consumption [30].

The presence of a caregiver (AOR 5.83 [1.39–24.38]) and presence of family history of cognitive impairment (AOR 7.94 [1.18–53.13]) were found to be significantly associated with the presence of MNCD in the multivariable analysis. The probable genetic predisposition for dementia has also been identified in other studies [24]. There is also a possibility that the patients or informers of patients with dementia may not have been able to accurately remember whether their parents or other family members may have had any degree of cognitive impairment. Patients with MNCD also had impairment in their ADL and hence had a greater need for a caregiver to help them with their daily needs.

In the bivariate analysis, female gender (1.93 [1.07–3.48]), no formal education (2.11 [1.23–3.62]), belonging to BG Prasad socio‐economic Class III or lower (1.89 [1.00–3.57]), presence of cognitive impairment in the family (5.42 [1.89–15.54]), and presence of vascular disease in the study participant (2.68 [1.37–5.28]) were found to be significantly associated with the presence of depression in the participant. These findings were similar to the factors found to be associated with depression in similar studies [31, 32, 33]. Out of these, presence of vascular disease (AOR 2.42 [1.18–4.98]) and presence of cognitive impairment in the family (AOR 4.25 [1.39–12.95]) were significant in the multivariable analysis, whereas female gender, no formal education, and lower socio‐economic status were not significant on adjusting for covariates.

Although the above findings are limited to one rural block, cognitive impairment is an ever‐present looming threat to the elderly and their health. With the prevalence of cognitive impairment found to be similarly high in other settings [18, 20], there is a need to identify strategies to screen for cognitive impairment in the existing community health systems. There are concerns regarding multiple languages, varying education status, and caregiver reporting of cognitive functions, which make implementation of a large‐scale screening program difficult [31]. However, factors found to be associated with cognitive impairment in this study, such as age greater than 70, having no formal education, and lower socio‐economic status, could be corroborated and confirmed in other studies in different settings and serve to inform specific strategies towards those at a higher risk of developing cognitive impairment. Existing rural primary care and community health systems already have programs aimed at screening the elderly for varied health problems such as diabetes and hypertension among others. Screening for cognitive impairment could be seamlessly included among these existing programs and help combating the effects of cognitive impairment.

## 5 Limitations

There is a possibility of inaccurate recall by the study participants or their caregivers leading to information bias. This affected questions regarding subjective cognitive decline, previous history of head injury, family history of cognitive impairment, and family history of psychiatric illness. This study was done in a cross‐sectional manner, and hence variables whose lifetime exposure affects the presence of cognitive impairment such as diabetes, hypertension, smoking, and alcohol consumption could not be accurately studied. Causality also cannot be established with regard to certain associated factors due to the inherent nature of the cross‐sectional study. Longitudinal studies done in the future could be essential to establish causality and strengthen the findings of this study.

The study was done in one rural block in Tamil Nadu, hence the broader applicability of some of these findings need to be confirmed further with studies in other areas.

The MoCA has the potential to be influenced by the education status of the participant, as it was initially designed in a setting with higher education levels than this study setting. This was countered by using a cut‐off more appropriate for the current study. However, there is still a possibility of certain participants being considered to have objective cognitive decline due to low scores in the MoCA test as a result of having no formal education but meeting no other criteria for cognitive impairment.

Lower case numbers among those with MNCD limit the reliability of odds ratios and may result in wide confidence intervals in certain bivariable analysis.

Depression was assessed using the GDS‐15 which is a screening tool and does not provide a conclusive diagnosis of depression. Furthermore, the presence of cognitive impairment may have affected the responses provided by the participants for the questions in the Geriatric Depression Scale.

## 6 Conclusions

Cognitive impairment is an emerging public health problem that needs to be studied in depth in order to improve our understanding of the condition and alleviate the suffering of the individuals with cognitive impairment. This study shows the ever‐present burden of cognitive impairment in Kaniyambadi block, Vellore, the different stages of cognitive impairment in the population, and the factors associated with it. Future studies could focus on these aspects and establish causality in order to formulate prevention and screening strategies for the early diagnosis of cognitive decline in older adults.

## Author Contributions

**Manoj Jacob Dhinagar**: conceptualization, investigation, writing – original draft, methodology, software, formal analysis, validation, data curation, funding acquisition, visualization. **Vinod Joseph Abraham**: conceptualization, methodology, writing – review and editing, supervision, validation, project administration, data curation. **Zacharia Mathew**: conceptualization, methodology, writing – review and editing, project administration, resources, supervision.

## Ethics Statement

Approved by the Institutional Review Board, Christian Medical College (IRB Min NO 14374, December 8, 2021) and the study adhered to their guidelines.

## Consent

Informed consent was obtained from every study participant by the authors.

## Conflicts of Interest

The authors declare no conflicts of interest.

## Data Availability

Data can be obtained from the corresponding author based on a credible request.
